# Correction: Protective porcine influenza virus-specific monoclonal antibodies recognize similar haemagglutinin epitopes as humans

**DOI:** 10.1371/journal.ppat.1009815

**Published:** 2021-08-04

**Authors:** Barbara Holzer, Pramila Rijal, Adam McNee, Basudev Paudyal, Veronica Martini, Becky Clark, Tanuja Manjegowda, Francisco J. Salguero, Emily Bessell, John C. Schwartz, Katy Moffat, Miriam Pedrera, Simon P. Graham, Alistair Noble, Marie Bonnet-Di Placido, Roberto M. La Ragione, William Mwangi, Peter Beverley, John W. McCauley, Rodney S. Daniels, John A. Hammond, Alain R. Townsend, Elma Tchilian

In Fig 6, data points from Fig 6B BAL-4DPI graph were inadvertently reused in Fig 6B NS - 1DPI instead of relevant data points when measuring virus titer in respective tissues. Please see the correct Fig 6 below.

**Fig 6:**
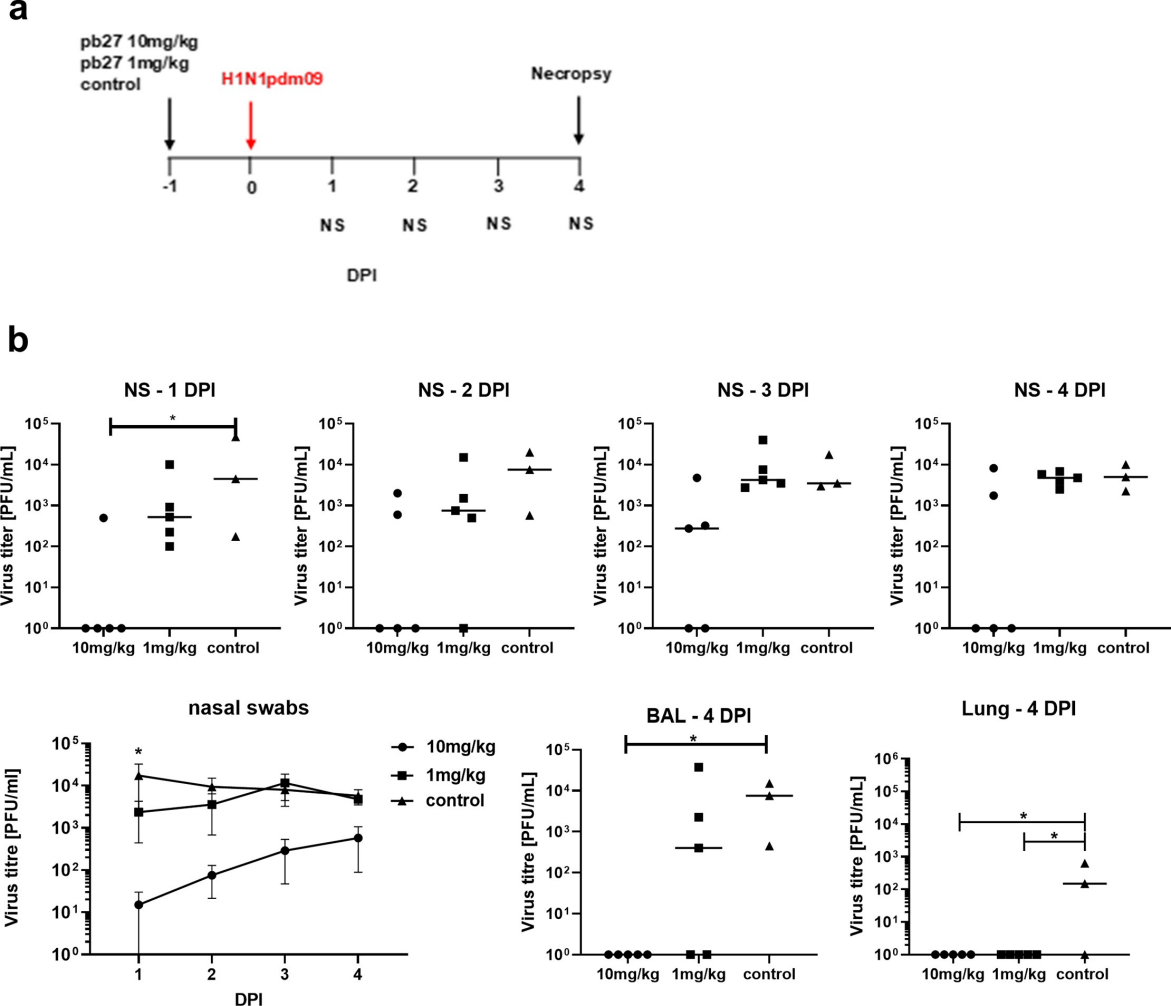
*In vivo* efficacy of pb27. pb27 was administered intravenously to pigs at 10 mg/kg and 1mg/kg, which were infected with H1N1pdm09 virus 24 hours later. Only three control animals were available. Nasal swabs (NS) were taken daily post-infection (DPI) and pigs euthanized at 4 DPI (**a**). Virus titers in NS, accessory lung lobe (Lung) and BAL at 4DPI were determined by plaque assay (**b**). Each data point represents an individual pig within the indicated group and bars show the mean. Virus shedding in NS is also represented as the mean of the 5 pigs on each day and significance versus diluent control indicated by asterisks. Virus titers were analysed using one-way non-parametric ANOVA, the Kruskal-Wallis test. Asterisks denote significant differences *p<0.05 versus control.
