# Supplementary material for: Protective porcine influenza virus-specific monoclonal antibodies recognize similar haemagglutinin epitopes as humans
Source: PLoS Pathog. 2021 Mar 4;17(3):e1009330. doi: 10.1371/journal.ppat.1009330 (PMC7932163; doi:10.1371/journal.ppat.1009330)
Supplement: S1 Fig — Samples were gated on lymphocytes (SSC-A vs. FSC-A) and singlets (FSC-H vs. FSC-A), live cells were identified as negative for live dead stain. HA-specific IgGHI cells were then identified as CD3-, CD8α-, CD172α- and double positive for HA and IgG. Non-specific binding was controlled by gating on mesenteric lymph node cells. (DOCX) [file ppat.1009330.s002.docx]

Lymphocytes live


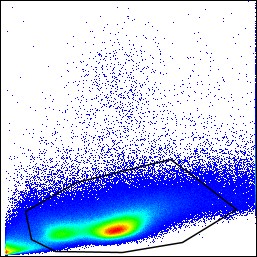

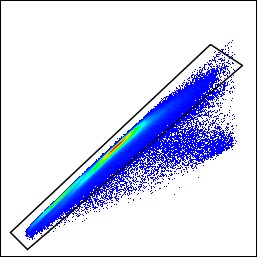

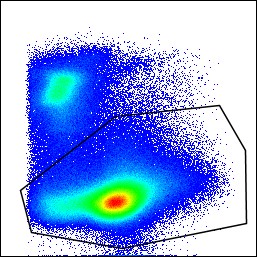


FSC

-

A

SSC

-

A

FSC

-

H

FSC

-

A

FSC

-

A

Live dead NIR

Lineage negative HA^+^ IgG^HI^ HA^+^ IgG^HI^


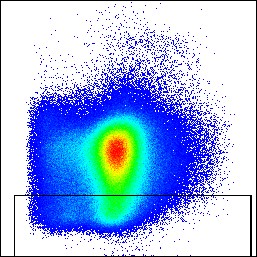

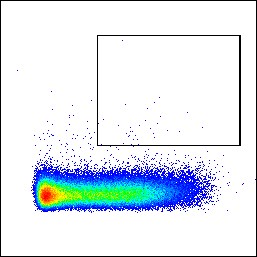

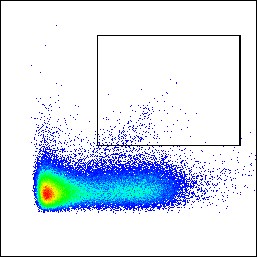


CD3, CD8

α

, CD172

α

FSC

-

A

IgG

HA

IgG

HA

0.04

0.31

Mesenteric LN Lung LN

**S1 Fig: Gating strategy for isolating single HA specific antibody producing cells.** Samples were gated on lymphocytes (SSC-A vs FSC-A) and singlets (FSC-H vs FSC-A), live cells were identified as negative for live dead stain. HA-specific IgG^HI^ cells were then identified as CD3-, CD8a-, CD172a- and double positive for HA and IgG. Non-specific binding was controlled by gating on mesenteric lymph node cells.
